# Regulation of Tubulin Gene Expression: From Isotype Identity to Functional Specialization

**DOI:** 10.3389/fcell.2022.898076

**Published:** 2022-05-26

**Authors:** Ivana Gasic

**Affiliations:** Department of Molecular and Cellular Biology, University of Geneva, Geneva, Switzerland

**Keywords:** tubulin, isotype, microtubule, transcription, autoregulation

## Abstract

Genomes of higher eukaryotes encode a large tubulin gene superfamily consisting of at least six α and six β-tubulin isotypes. While some α and β-tubulin isotypes are ubiquitously expressed, others are cell-type specific. The subset of α and β-tubulins that is expressed in a given cell type is defined transcriptionally. But the precise mechanisms of how cells choose which α and β isotypes to express and at what level remain poorly understood. Differential expression of tubulin isotypes is particularly prominent during development and in specialized cells, suggesting that some isotypes are better suited for certain cell type-specific functions. Recent studies begin to rationalize this phenomenon, uncovering important differences in tubulin isotype behavior and their impact on the biomechanical properties of the microtubule cytoskeleton. I summarize our understanding of the regulation of tubulin isotype expression, focusing on the role of these complex regulatory pathways in building a customized microtubule network best suited for cellular needs.

## Introduction

A vast diversity of subcellular architectures exists in nature. One prominent example is the cytoskeleton found in various arrays not only across the different cell types but also within the same cell type and over the course of the cell cycle. The morphological diversity tangoes with functional specialization. This is acutely evident for one group of cytoskeletal elements—the microtubules. Microtubules are dynamic polymers of α and β-tubulin isotypes, which carry out a number of diverse functions in cells, including flagellar motility, intracellular transport, chromosome segregation, and the establishment and maintenance of cellular morphology (Muroyama and Lechler, 2017). How do eukaryotic cells create such a spectacular diversity in form and function with a set of presumably uniform building blocks? It is well established that a plethora of tubulin and microtubule associated proteins (MAPs) are able to shape the biomechanical properties of microtubules, thus introducing a sophisticated layer of regulation and functional specialization (Goodson and Jonasson, 2018; Cuveillier et al., 2020). But in principle, alterations in the properties of tubulins themselves could both directly and indirectly (through MAPs) affect the assembly and biomechanical properties of microtubules. This idea, first articulated as the multitubulin hypothesis, is based on biochemical differences observed among tubulins isolated from single species and the discovery that most eukaryotic cells express multiple isotypes of α and β-tubulin proteins (Fulton and Simpson, 1976; Stephens, 1978; Cleveland et al., 1980). While the influence of MAPs on microtubule network morphology and function has been reviewed elsewhere (Goodson and Jonasson, 2018), I focus on the role of the regulation of α and β-tubulin gene expression in eukaryotic cells. I refer to isotypes as tubulin species arising from multiple genes and refrain from addressing their posttranslational modifications, which others have thoroughly discussed (Magiera and Janke, 2014; Yu et al., 2015; Gadadhar et al., 2017; Roll-Mecak, 2020; Guichard et al., 2021). Finally, I review some examples of the functional specializations of isotypes and raise the question of why cells evolved such a high complexity of tubulin gene networks.

## Tubulin Isotype Expression and Functional Specialization

### Tubulin Superfamily and Transcriptional Regulation of Gene Expression

**FIGURE 1:**
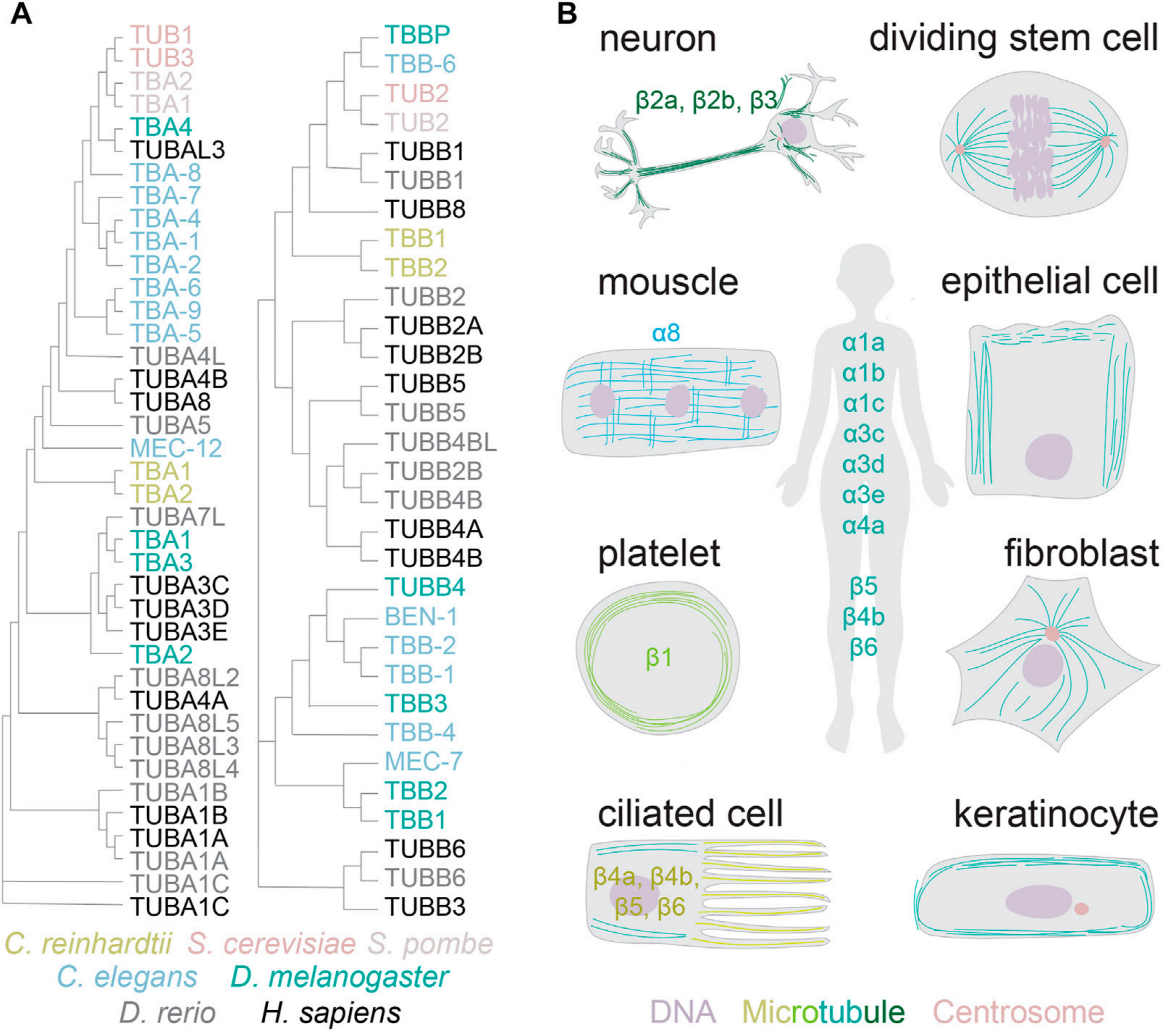
Tubulin gene networks and protein distribution. **(A)** Phylogenetic neighbour-joining tree without distance corrections (with cladogram branch length, created with Clustal Omega). Color coded are tubulins from the species referenced in this review article. **(B)** The distribution of tubulin isotypes and diversity of microtubule architectures across specialized cells in higher eukaryotes.

Genomes of higher land plants and metazoans encode the core microtubule proteins, α and β-tubulins, in large multi-gene families (Figure 1A) (Gasic and Mitchison, 2019). Unicellular eukaryotes, such as yeast and green algae, encode one to two different α and β-tubulin subunits. The complexity of tubulin gene networks increases with multicellularity: higher eukaryotes encode six to nine tubulin isotypes of each subunit (MacRae and Langdon, 1989). The isotypes share up to 99% identity, with most differences residing in the carboxy-terminal tails (Sullivan, 1988). Most isotypes are constitutively expressed, such as α1a and β5 (Ludueña, 1997). Others are restricted to specific tissues and cell types (Figure 1B). Prominent examples include platelet-specific β1 or neuron-specific β3-tubulin (Ludueña, 1997; Breuss et al., 2017). It is generally assumed that transcriptional regulation defines the expressed tubulins for both the constitutive and specialized forms.

**FIGURE 2:**
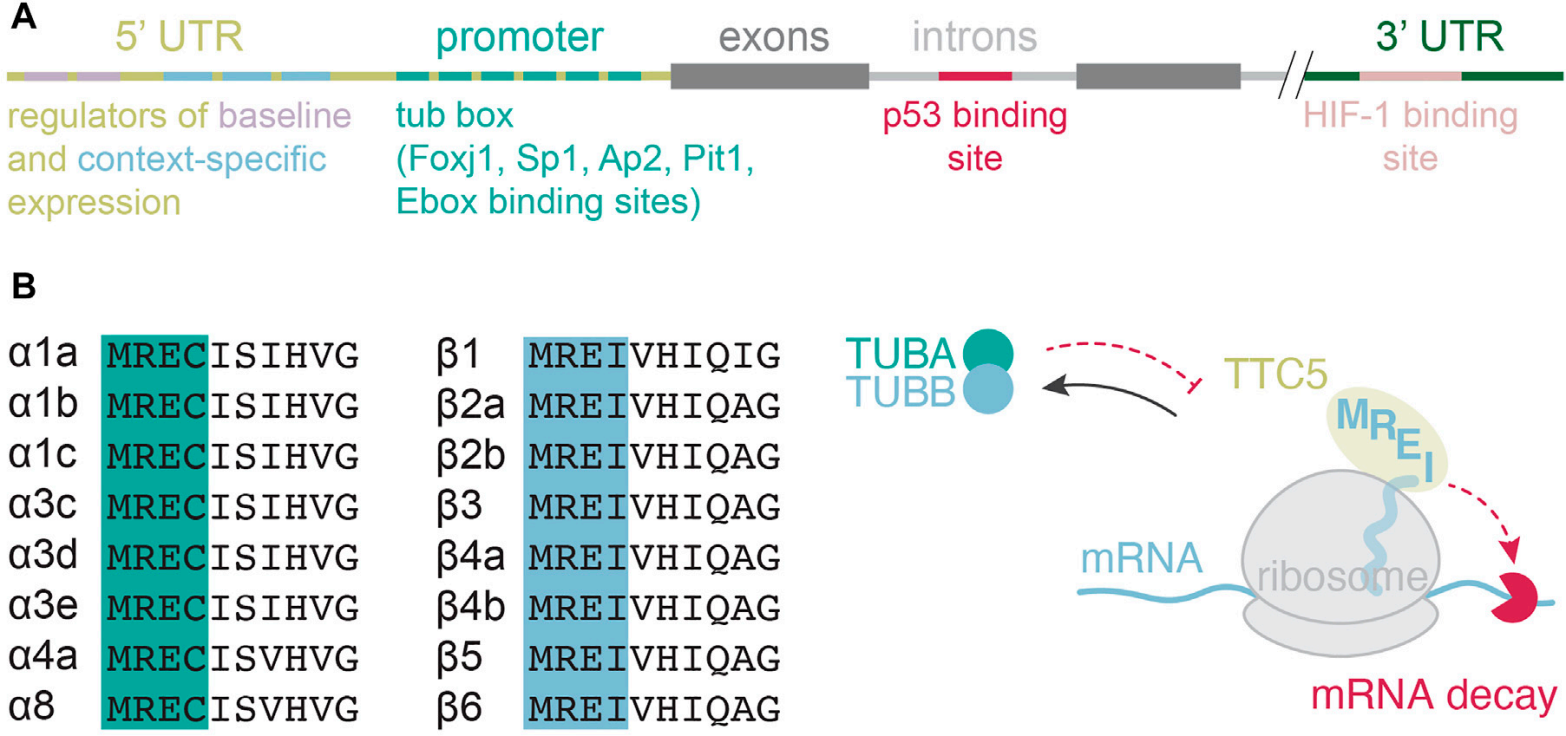
Transcriptional and posttranscriptional regulation of tubulin gene expression. **(A)** Transcriptional regulatory elements mapped in various isotubulin genes and across species. **(B)** Amino-terminal protein sequence alignment of isotubulins and the current model for tubulin autoregulation.

In most cell types, tubulin gene transcription is considered a part of a general “housekeeping” program. But the mechanisms behind it remain poorly understood. For instance, transcriptional factors that may drive such constitutive expression remain largely elusive. Numerous regulatory regions in tubulin genes have been found (Figure 2A), but the precise mechanisms of how they orchestrate tubulin gene transcription have been investigated only in some specific contexts. Four main complications challenge studies of tubulin gene transcription. First, most cells express multiple tubulin genes, subsets of which differ from one cell type to another. Hence, lessons learned from one cell type or one subset of tubulin isotypes do not apply to another one. Second, this intricacy is further exacerbated by the complexity of interacting partners and functions that tubulins perform (Muroyama and Lechler, 2017). Third, the encoded gene products are highly similar making them difficult to distinguish in downstream applications. And fourth, most of the field has historically been focused on using immortalized, fibroblast-like two-dimensional cell cultures to study tubulin biology. Such cells are not only taken out of their natural context, but also largely simplified systems that have hence lost their “identity” when it comes to tubulin gene expression regulation and microtubule function. The availability of complex model systems that better represent nature, such as organoid cultures, as well as sophisticated genetic engineering, immunolabeling, and genome-wide transcriptomic analyses should facilitate progress in identification and characterization of the transcriptional networks that govern constitutive tubulin gene expression.

Transcriptional regulation of tubulin genes is much better understood in conditions where tubulin is required to build specialized assemblies. One prominent example is a burst in tubulin gene transcription during flagellar regrowth following deflagellation in unicellular green algae *Chlamydomonas reinhardtii*. *C. reinhardtii* possesses four tubulins called α1, α2, β1, and β2-tubulin, encoded by four distinct genes. Rapid flagellar excision induced by mechanical stress or an acid shock triggers a coordinated transcription of a set of flagellar genes including tubulins. This robust response has been used to study the principles governing transcriptional control of the activated tubulin genes, leading to the discovery of the first tubulin gene promoter—the β2 (Davies et al., 1992; Davies and Grossman, 1994). The identified promoter contains seven repeats of a 10-base pair (bp) motif, named tub box, between the TATA box and the transcription initiation site (Figure 2A). The tub box motifs are required for β2-tubulin gene transcription following deflagellation. Similar investigations aimed at identifying the regulatory elements in the α1-tubulin gene revealed yet higher complexity with two promoter regions that act as regulatory elements (Figure 2A). The upmost region (-176 to -122 bp) emerged as a regulator of baseline transcription, keeping the expression levels low. The second region (-85 to -16 bp) encodes an activator for deflagellation-induced gene expression (Periz and Keller, 1997). Recent genetic studies identified two paralogous Foxj1a and Foxj1b transcription factors as core drivers of the motile ciliogenic program in the zebrafish embryo (Yu et al., 2008). Foxj1a turns on a set of genes required for the formation and function of motile cilia. The paralogous foxj1b appears to regulate motile cilia formation in the otic vesicle. Curiously, foxj1a is not required for the formation of primary cilia, which are immotile and involved in signaling (Yu et al., 2008). This finding further supports the idea that transcriptional programs that control the expression of tubulins and related genes are highly function-and context-dependent.

In the fruit fly *Drosophila melanogaster,* β2-tubulin isotype is expressed exclusively during spermatogenesis. The β2-tubulin promoter element responsible for the tissue specific gene expression is spread over a region of 80 bp and is sufficient to drive germline-specific expression in the testis (Michiels et al., 1989). An additional 14 bp activator element called β2UE1 confers promoter specificity to spermatogenesis (Santel et al., 2000). Equally complex regulatory elements are likely to be present and govern the expression of other tissue-specific tubulins. For instance, the neuron-specific α1-tubulin gene promoter contains a conserved repetitive homeodomain consensus sequence core (TAAT) with a flanking basic helix—loop–helix binding enahancer box (E-box) (Hieber et al., 1998; Goldman and Ding, 2000). While these elements are not essential for α1-tubulin gene transcription in the zebrafish retinal ganglion cells during development or in regenerating retina after neuronal damage, they are necessary for induced gene transcription in response to optic nerve crush (Senut et al., 2004). The neuron-specific β countertype is β3-tubulin, whose start site and the TATA box at position -28 bp are similar to other tubulin genes. The promoter region of β3-tubulin gene encodes numerous putative binding sites for specificity protein 1 (Sp1), Activating Enhancer Binding Protein 2 (AP2), pituitary-specific transcription factor 1 (Pit1), or Ebox (Figure 2A) (Dennis et al., 2002). But how these binding sites are engaged to drive neuron-specific expression of β3-tubulin remains to be elucidated.

In addition to the elements upstream of transcription initiation site, some regulatory sequences are also found in introns of tubulin genes (Figure 2A). For instance, the first intron of mouse β2-tubulin gene contains a p53 binding site, which acts as a silencer for β2-tubulin transcription. Antagonizing the binding of p53 induces gene expression (Arai et al., 2006). The discovery of this mechanism rationalized resistance of a mouse melanoma cell line B16F10 to vinca alkaloids: vinca alkaloids prevent p53 binding to the intronic regulatory element thus driving overproduction of β2-tubulin (Arai et al., 2006). In the case of the *Drosophila* β3-tubulin gene, constitutive expression is achieved through at least two cis-acting elements, upstream and downstream of the transcription initiation site (Gasch et al., 1989), but regulatory elements in the intron mediate transcription in a tissue-specific manner (Gasch et al., 1989). Two opposing factors recognize the intronic regulatory elements to balance gene transcription: the ultrabithorax (Ubx) driving transcription of β3-tubulin gene, and the engrailed (En) repressing it (Serrano et al., 1997).

Finally, additional transcriptional regulatory sequences have been found in the 3′ untranslated regions of tubulin genes (UTR, Figure 2A). One such example is the hypoxia inducible factor 1 (HIF-1) binding site present in the 3′-flanking region of human β3-tubulin gene, which is thought to drive ectopic β3-tubulin expression in tumors (Raspaglio et al., 2008). In healthy tissues, β3-tubulin is restricted to neuronal and Sertoli cells (Easter et al., 1993), and during defined periods of development (Katsetos et al., 2003), indicating the existence of spatio-temporal clues governing its expression. But tumor cells are frequently found to abnormally express β3-tubulin and in significantly larger quantities (reviewed in (Drukman and Kavallaris, 2002)). In tumors, the expression levels of β3-tubulin correlate with poor prognosis, indicating that not only the isotype composition but also protein levels are relevant. How tumor cells lose the breaks and enter β3-tubulin overproduction remains unknown. In general, transcriptional networks and regulated proteolysis together set protein abundances. But for tubulins in higher eukaryotes, a posttranslational mechanism called tubulin autoregulation is thought to act as an additional tailor of protein levels.

### Tubulin Autoregulation

Tubulin autoregulation is a general mechanism that operates on all α and β-tubulin isotypes (Figure 2B) and in all higher eukaryotic cells tested so far (Gasic and Mitchison, 2019; Fellous et al., 1982; Pittenger and Cleveland, 1985; Caron et al., 1985; Lau et al., 1986; Cleveland, 1989). When in excess of cellular needs, tubulin proteins negatively regulate the stability of their encoding mRNAs (Ben-Ze’ev et al., 1979; Cleveland et al., 1981). This negative feedback mechanism requires an ongoing translation (Gay et al., 1989), as cells use tubulin nascent proteins to recognize tubulin mRNAs and target them for degradation. The recognition motif resides in the first four amino-acids of nascent α and β-tubulins (Theodorakis and Cleveland, 1992; Yen et al., 1988; Bachurski et al., 1994), and is recognized by tetratricopeptide protein 5 (TTC5), which acts as the specificity factor in tubulin autoregulation (Figure 2B) (Lin et al., 2020).

Pioneering studies of tubulin autoregulation failed to resolve the regulation of individual tubulin subunits due to technical limitations, such as lack of tubulin isotype specificity probes. Recent efforts deployed reverse transcription-based quantitative polymerase chain reaction and transcriptomics, offering a much higher resolution of individual isotype regulation. These studies reveal that all the expressed α and β-tubulins are subject to autoregulation in higher eukaryotes (Gasic et al., 2019). This is perhaps not surprising, given the mechanistic dependence of tubulin autoregulation on the nascent tubulin tetrapeptide sequences and their high conservation (Figure 2B).

While all the tubulin isotypes are subject to posttranscriptional regulation in higher eukaryotes, the extent to which they are autoregulated varies within the same cell type and across the different tissues (Gasic et al., 2019). The most likely explanation is that these differences stem from varying rates of isotubulin translation—the more a certain mRNA species is translated, the higher the rate of mRNA decay in tubulin autoregulation. For instance, β3-tubulin mRNA appears to be less regulated even though reasonably abundant in human cultured cells. It is possible that β3-tubulin mRNA is little or not translated in cultured cells, especially given that β3-tubulin isotype is neuron specific. Further studies are required to evaluate the level of β3-tubulin mRNA autoregulation in neurons and cancer tissues where its expression is elevated. Likewise, whether cells translate tubulin isotypes at different rates remains unknown. The availability of ribosome footprint profiling technologies should facilitate progress in this direction. An alternative explanation is that the observed differences in autoregulation between tubulin isotypes are not real, but rather a technical artifact, where transcripts present in very small amounts may falsely show large fold changes in abundances across samples. For instance, β2b-tubulin mRNA is present at very low levels and may not be regulated at all in cultured cells (Gasic et al., 2019). Remarkably, in neurons, β2b-tubulin mRNA appears to be chaperoned by the microtubule plus-end-tracking protein adenomatous polyposis coli (APC). APC associates with the 3’ UTR of β2b-tubulin bringing it into the growth cone, where it is translated and the protein incorporated into microtubules (Preitner et al., 2014). Whether APC or other mRNA binding proteins may act to protect specific tubulin mRNAs from autoregulation or modulate their rate of decay remains to be elucidated. More generally, it remains to be seen whether tubulin autoregulation can further exacerbate the differences in tubulin isotype levels in cells and to what extent.

Collectively, transcriptional regulation and autoregulation paint a picture in which the different tubulin isotypes are specifically expressed at different levels. Why do cells recruit such complex cellular machineries to provide differential expression of tubulin isotypes? A growing body of evidence points toward functional diversification and specialization of tubulin isotypes, some examples of which I discuss further.

### Functional Specialization of Tubulin Isotypes

Initial analyses of tubulin mutations, gene disruption, introduction of chimeric genes, and immunolabeling of endogenous tubulins to visualize the distribution in cells revealed that tubulin isotypes are largely interchangeable (Bond et al., 1986; Joshi et al., 1987; Lewis et al., 1987; Lopata and Cleveland, 1987). These studies, however, used exclusively the divergent carboxy-termini but no other regions to differentiate between the tubulin isotypes. Results of a deeper look at tubulin isotype properties and functions have not borne up to the original findings. A growing body of evidence suggests that tubulin isotypes carry inherent differences, conferring distinct architectures and biomechanical properties to microtubules (Lu and Luduena, 1994; Panda et al., 1994; Vemu et al., 2017). Such functional specialization is present already in yeast, where two α-tubulin isotypes show opposing effects on microtubule dynamics *in vitro*, and a biased affinity towards the spindle positioning machinery during yeast mitosis (Bode et al., 2003; Nsamba et al., 2021).

Perhaps the most peculiar tubulin assemblies are axonemes. Localized at the center of cilia and flagella, axonemes provide these subcellular compartments structural integrity, mobility, and mediate transport and signaling. Axonemes emanate from centrioles—a pair of cylindrical structures composed of nine triplet microtubules organized in a radially symmetrical array (Guichard et al., 2021). This 9-fold radial symmetry carries over into the axoneme, albeit not as triplet but as doublet microtubules. An additional central pair of parallel microtubules is seen in motile cilia. The central pair and transition from triple to doublet organization are not sole differences between centrioles and axonemes: while the fruit fly β1-tubulin isotype dominates centriolar microtubules, the β2 isotype dominates the axonemal ones (Nielsen et al., 2001). This specificity in tubulin isotype composition has been studied mainly in *Drosophila melanogaster* and appears to be critical for centriole and axoneme formation and function. In fruit fly male germ line, the β1-tubulin alone cannot function in axonemes (Raff et al., 2000). These males form significantly shorter axonemes without the central pair of microtubules. Similarly, when β1-tubulin exceeds β2, the axonemes contain 10 instead of nine doublets in addition to promiscuous axoneme formation in the cytoplasm. An abnormal expression of β3-tubulin disrupts the assembly of microtubule doublets (Hoyle and Raff, 1990). In addition to β2, mammalian cilia also contain substantial proportion of the β4-tubulin isotypes a and b, both of which contain axoneme-specific carboxy-terminal motifs (Jensen - Smith et al., 2003). It remains to be elucidated how the tubulin isotype composition impacts the axoneme architecture and function. One tempting explanation lies in the interaction with the other structural and functional components of the axonemes. For instance, the motor proteins responsible for cargo transport inside the axoneme may have a preference for walking on certain tubulin isotypes (Jordan et al., 2018).

Another example of highly specialized microtubule network is seen in platelets, where microtubules are organized as a circumferential ring known as the marginal band (Figure 1). Marginal bands provide structural integrity and the typical discoidal shape to platelets. Several studies estimate β1-tubulin to comprise the majority of total β-tubulin in these cells (Lewis et al., 1987; Burkhart et al., 2012). This highly divergent tubulin isotype confers curvature to the microtubules of the marginal band. It remains unknown whether this is a direct effect of tubulin conformation or an indirect effect through microtubule binding proteins. Ectopically expressed β1-tubulin drives the formation of curved cytoplasmic microtubules in other cell types, and confers resistance to microtubule destabilizing poisons (Yang et al., 2011). Curiously, marginal bands in avian red blood cells contain also β3-tubulin thought to facilitate microtubule assembly and resistance to cold-induced depolymerization (Murphy and Wallis, 1985; Murphy and Wallis, 1986; Joshi et al., 1987). The β3 isotype is, however, not required for the formation of the marginal band in these cells (Swan and Solomon, 1984). The molecular-level details of how these different isotubulins contribute to the organization of the marginal band remain to be uncovered. The availability of sophisticated tools for structural analyses should facilitate progress in understanding the microtubule cytoskeleton in these highly specialized structures, and promises to reveal interesting mechanisms that cells utilize to create pattern architectures.

Molecular studies in the worm *Caenorhabditis elegans*, also indicate that particular tubulin isotypes can infiuence the supramolecular organization of microtubule lattices. For instance, the β-tubulin isotype MEC-7 from *C. elegans* is expressed primarily in microtubules within the axons of touch receptor neurons (Hamelin et al., 1992). Although worm microtubules normally consist of 11 protofilaments, the touch receptor axonal microtubules are structurally distinct and consist of 15 protofilaments. The MEC-7-null mutants, however, form axonal microtubules based on 11 protofilaments, indicating that the MEC-7 isotype specifically infiuences the architecture of axonal microtubules (Savage et al., 1994). The α-tubulin MEC-12 is also required for 15-protofilamtent microtubule assembly (Fukushige et al., 1999). Loss of either MEC-7 or 12 leads to touch insensitivity (Savage et al., 1994).

In addition to structural changes in the microtubule network, tubulin isotype composition has been found to influence microtubule biomechanical properties. One such example are microtubules in neurites. During neurite extension, cells quadruple the expression of β2 and β3 isotubulins and double the expression of β1-tubulin isotype. The expressed tubulins are incorporated into neurite microtubules in different proportions: while β2-tubulin dominates the neurite microtubules, β1, β3, and β4-tubulin isotypes are present in smaller quantities, and β5-tubulin is partially excluded (Joshi and Cleveland, 1989). Neurite microtubules are known to be substantially more stable than those found in cell bodies. The specific neurite isotubulin composition may provide structural stabilization within the microtubule lattice. *In vitro* studies with purified tubulins and in the absence of MAPs show that microtubules assembled from β2 or β4 isotypes are considerably less dynamic than those assembled of the neuron-specific β3-tubulin (Lu and Luduena, 1994; Panda et al., 1994; Pamula et al., 2016; Vemu et al., 2017). This finding is somewhat counterintuitive and suggests that potential other factors are deployed to stabilize neurite microtubules. During neurite outgrowth and concomitantly with the change in tubulin expression, cells begin to express the neuron-specific microtubule associated protein 2 (MAP2) and increase the expression of microtubule associated protein tau (MAPT) (Joshi and Cleveland, 1989). It is possible that MAP2 and MAPT bind the neurite microtubules with higher affinity, thus stabilizing them.

Protein regions that confer differences in the biochemical properties of tubulin isotypes remain elusive. Even though the most divergent and thus top candidates, the role of carboxy-terminal tails of tubulins in their dynamic behavior *in vitro* remains controversial (Pamula et al., 2016; Fees and Moore, 2018). Systematic studies *in vitro* and in cells are necessary to unambiguously elucidate the role of carboxy-terminal tails in regulating tubulin biochemical properties. In addition to carboxy-terminal tails, lateral contact interfaces also harbor large sequence variability between tubulin isotypes and are thus potential regulation sites for tubulins’ intrinsic biomechanical properties. High-resolution structures of isotypically pure microtubules may bring answers to how tubulin composition fine-tunes microtubule dynamics in cells. Recent structural analyses of *C. elegans* microtubules provide a proof of concept and reveal distinct features at lateral contact sites of tubulins likely responsible for the exceptionally high dynamic behavior of these microtubules (Chaaban et al., 2018).

## Discussion

Multi-gene families encode numerous important proteins, such as the histones, actins, various metabolic enzymes, or components of the immune system. Why does nature maintain multiple copies of genes that encode closely related proteins? For tubulins, the answer seems to reside in three independent but related aspects of gene function. First, the existence of multiple genes provides the opportunity for their differential expression throughout development and in specialized tissues. Various insults as well as physiological inputs trigger changes in tubulin gene expression. While some act purely via modulation of tubulin autoregulation, such as nutrient withdrawal, others such as amino-acid deficiency trigger transcriptional responses. In some instances, both regulatory mechanisms are engaged simultaneously (Gasic et al., 2019). We know very little about how all these pathways converge to provide cells with sufficient but not surplus tubulin. Is tubulin gene expression ever constitutive and as such at steady state? Or is it rather a dynamic result of a complex matrix of inputs that cells receive? If at any time a large number of inputs can modify tubulin gene expression, how do cells orchestrate their responses? It is tempting to speculate that the complexity in the number and nature of signals that shape tubulin gene expression may have been the primary drive for tubulin multiplication. Further investigations into how cells engage the elements in tubulin promoter regions to respond to various stimuli may provide some answers to these long-standing questions.

Second, the existence of multi-gene families allows diversification in the structures and functions of the encoded gene products. While we begin to unravel the differences amongst tubulin isotypes, a lot more work is warranted to provide a comprehensive view of their biochemical properties. Our understanding of how the different isotubulins are paired in heterodimers or how they are distributed in microtubule networks is rudimentary. Are some combinations of α and β isotypes favorable? How do their different combinations contribute to the biomechanical properties of microtubules? These questions are rather complex to study as there is a large number of possible combinations between the α and β isotypes further complicated by their spatial distribution in cells. Are some isotypes segregated in specific areas of the microtubule network? What would be the role of such an isotype code? In dynamic, short lived microtubules any information stored in this type of code would be quickly scrambled. But in long-lived microtubules, like those in neurons, spatial distribution of tubulin isotypes could encode information. To solve this puzzle, we need experimental systems that better represent nature and the diversity of microtubule architectures, as well as advanced tools to visualize individual tubulin isotypes.

Third, and much less discussed possible explanation is backup compensation, given that all tubulins can build microtubule networks capable of carrying out their most fundamental functions, such as cell division. In this concept, tubulin isotypes need not be discretely specialized. Rather, the focus is on conserved parts of the proteins. Differential expression of tubulin isotypes is then purely a consequence of the engagement of upstream transcriptional regulatory mechanisms that work to supply cells with tubulins of generalized function.

Most of the documented differences in isotype distribution across the different cell types and microtubule structures, as well as their functional specializations are related to β subunits. Are α-tubulins inherently more redundant? Do they harbor fewer differences and hence contribute less to the specialization of the microtubule cytoskeleton? Given fewer studies of α-tubulins it is difficult to answer these questions. Further studies may reveal new biology of α-tubulins or may reveal that they remain conserved. What would be further physiological implications of such different evolution of two proteins that form obligate heterodimers? And what can we learn about protein evolution from tubulins? These promise to be interesting areas of further exploration.

Careful analyses of tubulin gene expression at both transcriptional and posttranscriptional level are required and necessary to understand how cells define which tubulins to produce and in what quantities. Similarly, detailed studies of tubulin biomechanical properties are warranted to understand how the isotype composition fine-tunes microtubule dynamics. But more integrated approaches may be required to gain a comprehensive view of how this complex gene network is organized and deployed in various cell types.
